# Hair follicle stem cells and the collapse of self-tolerance in alopecia: the interplay of barrier function, the microbiome, and immunity

**DOI:** 10.1038/s44321-024-00170-7

**Published:** 2024-11-09

**Authors:** Joseph S Durgin, Sunny Y Wong

**Affiliations:** Department of Dermatology, Department of Cell and Developmental Biology, University of Michigan, Ann Arbor, MI 48109 USA

**Keywords:** Immunology, Skin, Stem Cells & Regenerative Medicine

## Abstract

J. Durgin and S. Wong discuss the article by Strobl et al, in this issue of *EMBO Mol. Med.*, that provides molecular insights and a therapeutic strategy for chronic folliculitis associated with EGFR-inhibitor anti-cancer therapy and cicatricial alopecia.

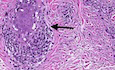

## Teaser

The hair follicle is thought to be a site of ‘immune privilege,’ or relative protection from autoimmune and inflammatory responses. In particular, the long-lived, quiescent hair follicle stem cells (HFSCs) are strikingly resistant to cytotoxic immune effectors such as T cells and natural killer (NK) cells, even in systems where these cells are artificially directed to attack HFSCs. Nonetheless, many forms of alopecia are associated with immune-mediated damage of the hair follicle epithelium, suggesting a failure of immune privilege. What precipitates the collapse of HFSC immune privilege in alopecia? In this current issue of *EMBO Molecular Medicine*, Strobl et al use an innovative mouse model to add to our mechanistic understanding of this disorder (Strobl et al, 2024).

The hair follicle is a dynamic miniature organ. Its permanent portion opens to the skin surface, communicates with sebaceous glands, harbors HFSCs in the bulge region, and receives the insertion of the arrector pili muscle. Its lower, non-permanent portion cycles between phases of telogen (resting), anagen (growth), and catagen (involution).

The phenomenon of immune privilege is associated with the downregulation of antigen presentation, upregulation of immune checkpoint molecules, and enrichment of immunosuppressive cell populations in the HFSC niche (Agudo et al, 2018; Cohen et al, 2024). That HFSCs have this protection, while stem cells in the intestine, ovary, and mammary glands do not (Agudo et al, 2018), makes it natural to wonder what special immunologic demands HFSCs face compared to stem cells in other organs. Certainly, the skin interacts with the external environment, and the hair follicle provides a portal of entry for both pathogens and commensal microbes, suggesting a possible evolutionary advantage for protecting HFSCs from an inflammatory milieu. The immune privilege may have also been a necessary evolutionary co-adaptation to allow for the hair follicle’s complex, cyclical mode of organ renewal.

**Figure 1:**
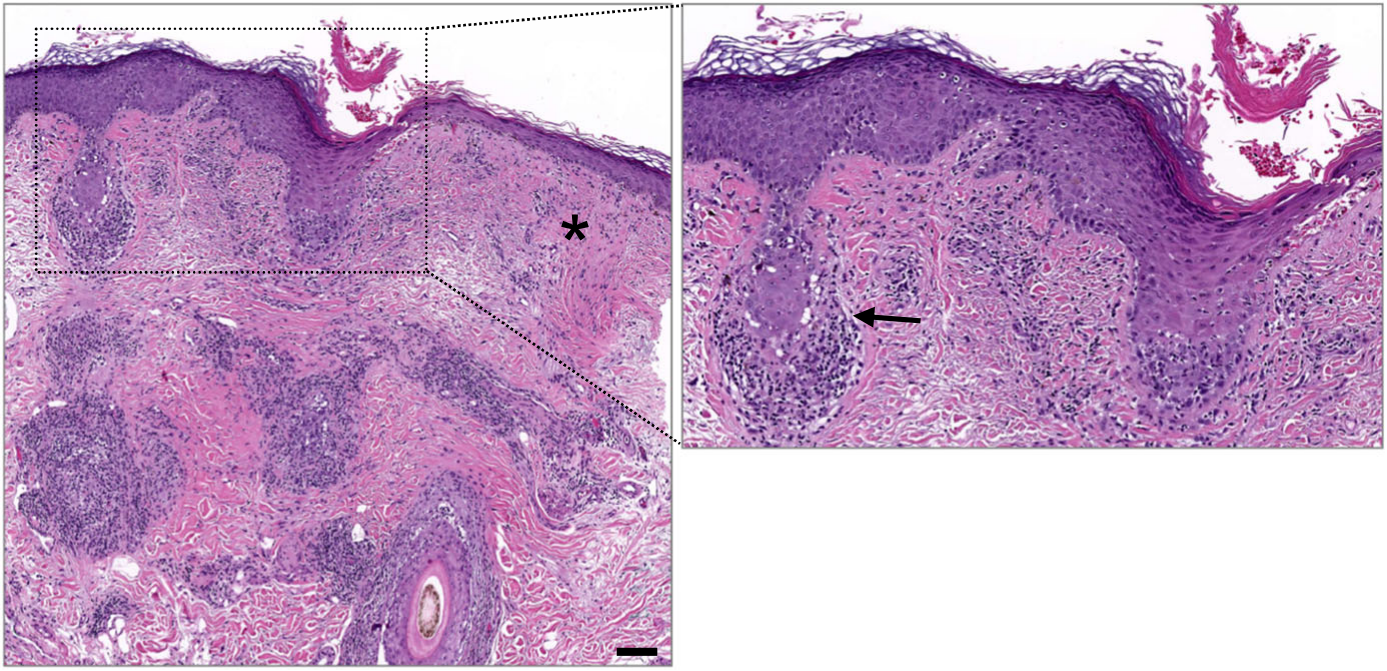
Histopathology of lichen planopilaris. Arrow, lymphocytic infiltration of the follicular epithelium with evidence of keratinocyte apoptosis. Asterisk, dermal fibroplasia and fibrous streamers replacing follicles. The follicular ostium shows hyperkeratosis. Scale bar, 50 μm. Image courtesy of Dr. Paul Harms.

Attack of the transient hair follicle bulb region by cytotoxic T cells results in reversible, non-scarring hair loss in alopecia areata (Gilhar et al, 1998). Autoimmune attack of the HFSC-containing bulge region, on the other hand, is thought to cause non-reversible, scarring (a.k.a. cicatricial) alopecia in diseases including lichen planopilaris (LPP) (Fig. 1) and discoid lupus erythematosus (Al-Refu et al, 2009; Harries et al, 2013). Therapies that target cytotoxic T cells are mainstays for treating both alopecia areata and scarring alopecia. Two Janus kinase (JAK)-inhibiting drugs, baricitinib and ritlecitinib, are now EMA-approved for alopecia areata, and the FDA has approved these and a third JAK inhibitor, deuruxolitinib. JAK inhibitors are now considered the first-choice therapy for alopecia areata, with glucocorticoids and oral minoxidil playing important adjuvant roles depending on the clinical setting (Rudnicka et al, 2024).

In the new study by Strobl et al, mice with hair follicle-specific deletion of epidermal growth factor receptor (EGFR^ΔEgr2^) acquire a phenotype of chronic folliculitis (inflammation around the follicle) that is associated with hair loss. These mice also exhibit a disrupted skin barrier, dysregulated microbiome, and upregulated JAK1/2 and STAT1 signaling caused by T cell- and NK cell-derived interferon (IFN)-γ. Four interventions in EGFR^ΔEgr2^ mice were each sufficient to prevent hair loss: treatment with an antibiotic to reduce microbial-induced inflammation, genetic inhibition of JAK-STAT signaling in the hair follicle, pharmacological treatment with a JAK inhibitor (ruxolitinib), and restoration of downstream EGFR signaling.

The EGFR^ΔEgr2^ model has several impressive symmetries with human disease. Patients treated with EGFR-inhibiting drugs (e.g., cetuximab or erlotinib) develop a pustular acneiform rash with the histopathologic features of neutrophilic folliculitis. These patients often have a good, albeit sometimes partial, response to antibiotics as well as to anti-inflammatory agents, including corticosteroids and topical JAK inhibitors. Strobl et al also demonstrate that two samples collected from patients with scarring alopecia show decreased expression of EGFR and its ligand AREG. These and additional samples from scarring alopecia patients showed increased interferon activity in keratinocytes, mirroring the findings in EGFR^ΔEgr2^ mice. Alopecia has also previously been reported in mice following targeted deletion of ADAM10, a protease involved with EGFR ligand shedding (Sakamoto et al, 2021). Whether EGFR deficiency is an inciting event in human alopecia, or merely a secondary (perhaps inflammation-driven) event that further sensitizes keratinocytes to interferon signaling, is not yet resolved by the available data.

Strobl et al further observe that the chronic folliculitis in EGFR^ΔEgr2^ mice eventually progresses to *scarring* alopecia, a phenotype also seen when *Pparg*, a master regulator of lipid metabolism, is deleted in HFSCs (Karnik et al, 2009). This distinction is clinically relevant, because hair loss disorders in dermatology are typically conceptually divided into scarring and non-scarring types. Because advanced cases of scarring alopecia are considered irreversible, those disorders often warrant a combination of systemic treatments to induce early remission. Therapies for end-stage scarring alopecia are wanting. Even hair transplantation in scarring disorders is controversial, because wound healing and graft survival in fibrotic tissues are unpredictable. The distinction is also mechanistically important, because scarring alopecia represents a more complete destruction of the HFSC niche. While fibrotic hair follicles and follicular plugging are observed in EGFR^ΔEgr2^ mice after 5 months of chronic folliculitis, the authors also observed impressive hair regrowth after treating the mice with topical ruxolitinib. The reversibility of the alopecia suggests that many chronically inflamed follicles in this model retain viability at 5 months. After 10 months, however, there is a decline in follicle number, and therapeutic intervention fails to restore hair growth. The progression of chronic folliculitis to irreversible alopecia suggests that the HSFC niche has either been destroyed or rendered permanently inactive after months of sustained injury.

Importantly, the loss of HFSC markers such as CD34 cannot be taken as proof that the stem cell niche is irreversibly damaged. Even at 5 months, hardly any CD34+ HFSCs were detected in EGFR^ΔEgr2^ mice. Nonetheless, with JAK inhibitor treatment, hair regrew and the HFSC niche was re-established. Therefore, the HFSCs were still recoverable despite the loss of CD34 expression at this timepoint. The correlation of multiple HFSC markers, or the use of genetic labeling methods to track cell fate, may help better characterize the loss or transformation of the HFSC niche during chronic folliculitis. Identifying biomarkers of stem cell viability in patients may allow for better stratification of potentially reversible stages of alopecia from permanent, end-stage disease.

Concordant with the findings of Strobl et al, recent studies have demonstrated that barrier disruption targeted specifically to the upper hair follicle is sufficient to cause hyperproliferation throughout the skin (Ford et al, 2024), and hair follicles are known to be active sites of cytokine secretion and immune cell recruitment (Ford et al, 2024; Sakamoto et al, 2021). In mice where EGFR is deleted throughout the epidermis during development, barrier defects do not manifest until hairs erupt (Klufa et al, 2019). Together, these studies support the concept that skin dysfunction might propagate from the hair follicle to the interfollicular epidermis in cutaneous disorders associated with barrier disruption.

The current study presents an elegant model in which barrier dysfunction, dysbiosis, and inflammation are conspiring insults that lead to chronic folliculitis and eventual permanent hair follicle destruction. Some critical questions remain. What specific microbial and/or self-antigens are responsible for the onset of inflammation in scarring alopecia? Are HFSCs the targets of immune cell-directed cytotoxicity, or are other modes of cell death operative? What are the disease-modifying roles of innate lymphoid cells, γδ T cells, regulatory T cells, and myeloid cells (Agudo et al, 2018; Cohen et al, 2024; Sakamoto et al, 2021)? Further studies using the valuable EGFR^ΔEgr2^ model will help untangle the complex mechanisms of stem cell dysfunction in scarring alopecia.

## References

[CR1] Agudo J, Park ES, Rose SA, Alibo E, Sweeney R, Dhainaut M, Kobayashi KS, Sachidanandam R, Baccarini A, Merad M et al (2018) Quiescent tissue stem cells evade immune surveillance. Immunity 48:271–285 29466757 10.1016/j.immuni.2018.02.001 PMC5824652

[CR2] Al-Refu K, Edward S, Ingham E, Goodfield M (2009) Expression of hair follicle stem cells detected by cytokeratin 15 stain: implications for pathogenesis of the scarring process in cutaneous lupus erythematosus. Br J Dermatol 160:1188–1196 19298282 10.1111/j.1365-2133.2009.09074.x

[CR3] Cohen JN, Gouirand V, Macon CE, Lowe MM, Boothby IC, Moreau JM, Gratz IK, Stoecklinger A, Weaver CT, Sharpe AH et al (2024) Regulatory T cells in skin mediate immune privilege of the hair follicle stem cell niche. Sci Immunol 9:eadh0152 38181095 10.1126/sciimmunol.adh0152 PMC11003870

[CR4] Ford NC, Benedeck RE, Mattoon MT, Peterson JK, Mesler AL, Veniaminova NA, Gardon DJ, Tsai SY, Uchida Y, Wong SY (2024) Hair follicles modulate skin barrier function. Cell Rep 43:114347 38941190 10.1016/j.celrep.2024.114347 PMC11317994

[CR5] Gilhar A, Ullmann Y, Berkutzki T, Assy B, Kalish RS (1998) Autoimmune hair loss (alopecia areata) transferred by T lymphocytes to human scalp explants on SCID mice. J Clin Investig 101:62–67 9421466 10.1172/JCI551 PMC508540

[CR6] Harries MJ, Meyer K, Chaudhry I, Kloepper JE, Poblet E, Griffiths CE, Paus R (2013) Lichen planopilaris is characterized by immune privilege collapse of the hair follicle’s epithelial stem cell niche. J Pathol 231:236–247 23788005 10.1002/path.4233

[CR7] Karnik P, Tekeste Z, McCormick TS, Gilliam AC, Price VH, Cooper KD, Mirmirani P (2009) Hair follicle stem cell-specific PPARgamma deletion causes scarring alopecia. J Invest Dermatol 129:1243–1257 19052558 10.1038/jid.2008.369 PMC3130601

[CR8] Klufa J, Bauer T, Hanson B, Herbold C, Starkl P, Lichtenberger B, Srutkova D, Schulz D, Vujic I, Mohr T et al (2019) Hair eruption initiates and commensal skin microbiota aggravate adverse events of anti-EGFR therapy. Sci Transl Med 11:eaax2693 31826981 10.1126/scitranslmed.aax2693

[CR9] Rudnicka L, Arenbergerova M, Grimalt R, Ioannides D, Katoulis AC, Lazaridou E, Olszewska M, Ovcharenko YS, Piraccini BM, Prohic A et al (2024) European expert consensus statement on the systemic treatment of alopecia areata. J Eur Acad Dermatol Venereol 38:687–694 38169088 10.1111/jdv.19768

[CR10] Sakamoto K, Jin SP, Goel S, Jo JH, Voisin B, Kim D, Nadella V, Liang H, Kobayashi T, Huang X et al (2021) Disruption of the endopeptidase ADAM10-Notch signaling axis leads to skin dysbiosis and innate lymphoid cell-mediated hair follicle destruction. Immunity 54:2321–2337 34582748 10.1016/j.immuni.2021.09.001 PMC8516731

[CR11] Strobl K, Klufa J, Jin R, Artner-Gent L, Krauß D, Novoszel P, Strobl J, Stary G, Vujic I, Griss J et al (2024) JAK-STAT1 as therapeutic target for EGFR deficiency-associated inflammation and scarring alopecia. EMBO Mol Med. 10.1038/s44321-024-00166-3 10.1038/s44321-024-00166-3 PMC11628629 39521937

